# Editorial for the Special Issue on IMCO 2019

**DOI:** 10.3390/mi11070684

**Published:** 2020-07-15

**Authors:** Tarik Bourouina, Xuming Zhang

**Affiliations:** 1 CNRS ESYCOM Lab., Université Gustave Eiffel, ESIEE Paris, 77454 Marne-la-Vallée, France; tarik.bourouina@esiee.fr; 2 Department of Applied Physics, the Hong Kong Polytechnic University, Hung Hom 999007, Hong Kong, China

This special issue is a collection of 12 technical papers and two reviews that are expanded into full-length articles from the conference abstracts of the 9th International Multidisciplinary Conference on Optofluidics (IMCO 2019) held in Hong Kong in 14–17 June 2019. The IMCO conference series is held annually to promote scientific exchange and collaboration among leading international researchers across cutting-edge research fields such as micro/nanofluidics, optical devices and systems, plasmonics and metamaterials, biochemical sensors and medical diagnosis, imaging and display, fabrication and integration, and energy and environment.

In this Special Issue, 10 papers present integrated, multifunctional physical and chemical sensors based on different platforms such as silicon gas cells [1], microchips [2,3], and various forms of optical fibers [4,5,6,7,8,9,10]. These studies well demonstrate the beauty of optofluidics by integrating the microfabricated structures and the micro-optics for high sensitivity and specificity of sensing. In other technical papers, one paper utilizes the microfluidic reactors for plasmonic photocatalytic water purification [11]; another paper investigates how the oil viscosity affects the droplet generation in a T-junction [12]. In the two review articles, one summarizes the recent development of phage-based electrochemical sensors in terms of immobilization protocols and electrochemical detection techniques [13], and the other reviews the latest progress of MEMS metasurface applications [14] in terms of tuning mechanisms, operation band and tuning speed.

An observation based on the papers in this special issue is that, after nearly two decades of development, the focus on optofluidics research seems to turn from the attempts of expanding the application areas back to those star applications that can make best use of the strength of optofluidics—the interplay of light and liquid/solid/gas media within a micro-/nanoscale space.

We would like to express our gratitude to all the authors for their great contributions to this special issue. Sincere appreciation should go to all the reviewers for their efforts and visions to ensure the quality of review articles. We are also indebted to the Editor, Ms. Mengdie Hu, for her enthusiasm on and long-standing support for this research area, and her timely and efficient editing work.
